# Minimally important difference in cost savings: Is it possible to identify an MID for cost savings?

**DOI:** 10.1007/s10742-020-00233-5

**Published:** 2021-01-07

**Authors:** Mary Dooley, Annie N. Simpson, Paul J. Nietert, Dunc Williams, Kit N. Simpson

**Affiliations:** 1 Departments of Health Science and Research and Healthcare Leadership and Management, College of Health Professions, Medical University of South Carolina, Charleston, USA; 2 Department of Public Health Sciences, College of Medicine, Medical University of South Carolina, Charleston, USA

**Keywords:** MID, Minimally important differences, Cost savings

## Abstract

As healthcare costs continue to increase, studies assessing costs are becoming increasingly common, but researchers planning for studies that measure costs differences (savings) encounter a lack of literature or consensus among researchers on what constitutes “small” or “large” cost savings for common measures of resource use.  Other fields of research have developed approaches to solve this type of problem. Researchers measuring improvement in quality of life or clinical assessments have defined minimally important differences (MID) which are then used to define magnitudes when planning studies. Also, studies that measure cost effectiveness use benchmarks, such as cost/QALY, but do not provide benchmarks for cost differences. In a review of the literature, we found no publications identifying indicators of magnitude for costs. However, the literature describes three approaches used to identify minimally important outcome differences: (1) anchor-based, (2) distribution-based, and (3) a consensus-based Delphi methods. In this exploratory study, we used these three approaches to derive MID for two types of resource measures common in costing studies for: (1) hospital admissions (high cost); and (2) clinic visits (low cost). We used data from two (unpublished) studies to implement the MID estimation. Because the distributional characteristics of cost measures may require substantial samples, we performed power analyses on all our estimates to illustrate the effect that the definitions of “small” and “large” costs may be expected to have on power and sample size requirements for studies. The anchor-based method, while logical and simple to implement, may be of limited value in cases where it is difficult to identify appropriate anchors. We observed some commonalities and differences for the distribution and consensus-based approaches, which require further examination. We recommend that in cases where acceptable anchors are not available, both the Delphi and the distribution-method of MID for costs be explored for convergence.

## Background

Concern about the growing spending trend in healthcare (Dieleman et al. 2020) has prompted clinical and health policy decision makers to continually assess benefits and value of new treatments and care processes with an objective to control costs without sacrificing quality of care (Blumethal and Abrams 2020; Baicker and Chandra 2020). Rising healthcare costs are a national problem, and as part of efforts to control costs, studies to assess the effect of systems changes on costs abound (Hong et al. 2020; Farford et al. 2019). However, few cost studies report a formal power analysis, and the literature is silent on questions related to the magnitude of cost. This may lead to inefficient study designs with excessive sample sizes. This happens if we use a cost measure that does not provide the maximum power to detect a difference; for example, using total cost over some time period, instead of disease specific cost, or if we fail to consider known sub-group differences in expected cost as part of the randomization or analysis plan. Furthermore, cost is a complex study variable, because it may be viewed from an organizational finance or accounting perspective (fixed and variable costs and budget impact) by some decision makers, or from an economic perspective (opportunity cost or cost effectiveness) by other stake holders. Thus, it may be important to specify an MID for cost in a study in such a way that has “face validity” as an MID of cost differences from both a financial management and an economics perspective.

We found no studies indicating how to determine “big” or “small” cost savings, even for common measures, such as hospital admissions or primary care visits. This absence of a common understanding about the magnitude of meaningful cost savings is detrimental to good planning for health care program evaluations, quality improvement assessments, and for randomized studies that assess the value (cost and consequences) of innovative health systems changes. If we do not know how big “Big” is, we may design studies that are either under powered or inefficient, neither of which is desirable. Developing a common understanding and language needed to discuss the concepts of magnitude related to cost of care is needed, because health policy research relies on statistical significance tests (*p* values or confidence intervals) to judge the likelihood that cost differences, associations, and effectiveness demonstrated in our policy studies are unlikely to be due to chance. In this paper, we will (1) discuss the methods described in the literature used to define MIDs, (2) use two common resource use categories (hospital admissions and outpatient visits) that are important drivers of cost in many studies as examples for applying MID approaches to cost data, (3) show how cost MIDs behave when used in power analyses to inform study planning, and (4) make recommendations for issues to be explored further as our understanding of the usefulness of MIDs for cost improvement.

### Literature of MIDs

The determination of a minimally acceptable difference for clinical measures can be easily assessed through repeated use and clinician experience from observations of the outcomes to identify what is clinically important. Other focus areas, such as quality of life improvement, use guides to judge effect magnitude. The concept of a minimal clinically important difference (MCID) was developed by Jaeschke, Singer, Guyatt to create interpretability of the change in score of Quality of Life (QOL) questionnaires (Jaeschke et al. 1989). A MCID, or as later referred to as minimally important difference (MID), is defined as the smallest difference perceived as beneficial that would result in a change of the patient’s management (Guyatt et al. 2002). MIDs are identified by three methods: (1) anchor-based, (2) distribution-based, and (3) consensus-based (Guyatt et al. 2002; King 2011).

The anchor-based method maps the relationship between the change in score of the inconclusive assessment (target) with an independent measure (anchor) that has an already established meaningfulness and an association with the target (Guyatt et al. 2002). The anchor seeks to quantify the changes in score into trivial, small, moderate, or large categories. However, one important point of this method is that it recognizes that the same absolute difference in score may have different meaning across different portions of the scale. As an example, a 10-point change from 20 to 30 is likely to mean something different to patients and clinicians than a 10-point change from 90 to 100. Thus, according to Guyatt, interpreting results in ways that consider the proportion of patients achieving the incremental benefit may be more important than simply comparing mean differences (Guyatt et al. 2002).

The distribution-based method of determining MID examines the relationship between the magnitude of effect and variability (Guyatt et al. 2002). Typically, this is expressed as a ratio called Cohen’s D, where the magnitude is within patient difference and variability is between patient variability for the control group at baseline, or the pooled variability of control and treatment groups at baseline (Guyatt et al. 2002; King 2011). There are two inherent limitations to this method to be considered. First, variability of a measure is different for each study, thus, effect sizes may not be comparable across different populations with varying levels of homogeneity (Guyatt et al. 2002). Second, the interpretability of an effect size, in terms of a fraction of a standard deviation, may not be easily understood by many practicing clinicians and may therefore lack face validity for clinical relevance. Cohen sought to address the latter limitation by suggesting that commonly observed study differences encompass ranges of 0.2 SD, 0.5 SD, and 0.8 SD for effects considered small, moderate, and large changes, respectively. There has been some discussion in the literature about the arbitrariness of these cut points. However, studies have provided evidence that suggest the plausibility of Cohen’s ranges and consistency of standard deviations and MID within the same instrument.

The final method for specifying a MID involves a consensus or Delphi approach using expert opinion. This approach was pioneered by the Rand Corporation in the 1950s, where researchers recognized that expert judgement is often needed to solve complex problems when a definitive conclusion is not obvious (Pill 1971; Okoli and Pawlowski 2004; de Villiers et al. 2005). The Delphi approach uses expert opinion refined through a series of rounds (King 2011). The objective of the method is to distill the judgement of a panel of selected experts in a field using a process that is minimally susceptible to bias from the experts’ personal characteristics, such as persuasiveness, perceived status and charisma (Okoli and Pawlowski 2004). The Delphi method is conducted anonymously using questionnaires sent by mail, e-mail, or fax. Responses are summarized and returned to the experts for re-evaluation, until consensus is reached, or until it becomes clear that experts truly differ on this issue. Expert panel reconsiderations under conditions of anonymity reduces bias that may occur in face-to-face discussions where dominant personalities within a group of experts may sway the group’s expressed opinion (Okoli and Pawlowski 2004). The anonymity used in the Delphi method reduces bias from dominant experts while capitalizing on their knowledge and insights through repeated rounds with new responses based on summaries. The iteration provides an opportunity for group members to provide feedback and explain their choices and for individuals to reevaluate their choice when given information provided by other experts (Okoli and Pawlowski 2004).

The use of MIDs for studies of patient-reported outcomes (PROs) has matured (Revicki et al. 2008). It is recommended that MIDs be based on responses and anchors that are correlated at > 0.30.

### Application of MIDs to cost data 

It is expected that MIDs may vary by context, and that a single MID may not be sufficient for all study applications. Further, it is recommended that a MID should be based on multiple approaches and triangulation of methods. It is also reported that different methods for estimating MIDs often converge, and that a Delphi process is employed to select MIDs that are relevant to a study (Revicki et al. 2008).

While any of the three approaches for the development of MIDs are accepted as being appropriate for informing the planning of studies using QOL and clinical assessment tools, it is not known whether these approaches could be translated into use for healthcare utilization and costs. To examine how MIDs behave for cost studies, we chose to use cost differences for hospital admissions and clinic visits as examples of high-cost and low-cost study outcomes. These costs were chosen because these two types of units are often the relevant resources on which interventions to reduce cost of care are focused. We used (unpublished) data from studies of actual patient cohorts and their recorded cost measures to specify MIDs and then used these MID specifications to examine the statistical power and sample size variability imposed by each MID definition.

We examined the literature of MIDs for PRO studies to identify relevant criteria for judging how our MIDs perform. Criteria discussions often stressed measurement validity markers (construct validity, responsiveness) that are not central issues for cost studies. Ideally a MID for cost would be: (1) constant across similar types of cost “drivers”; (2) relevant for a specific costing perspective, and (3) be stable over a reasonable cost horizon. However, the assessment of MIDs on these criteria is outside the scope of this study. So, instead of judging our MIDs against specific criteria, we used the convergent approach recommended by Revicki and colleagues (2008) combined with a pragmatic comparison on power and sample size. The literature of MIDs for PROs recommends the use criteria for choosing MIDs that: (1) are based on selection of relevant ranges that emerge when results are presented graphically; (2) weigh anchor-based results heavier that results from other methods; (3) seek convergence between methods; and (4) use a modified Delphi approach for development of consensus. The choice of describing MIDs as they behave with regard to statistical power and sample size was pragmatic based on relevance to the planning of cost studies of relevance to population health, health systems reengineering and quality improvement efforts. This focus is supported by the statement Revicki and colleagues (2008) for the use of MIDs for PRO research, that “MIDs are clearly useful for calculating statistical power and for determining sample sizes for clinical trials”.

## Methods

The hospital admission cohort consists of patients identified as having an opioid-related event treated in any hospital in a state over a 3-year period. The clinic visit cohort consists of cost data from outpatient visits incurred over 12 months for HIV-infected adolescents from 4 clinics in different states in the US. These data were de-identified and are part of ongoing exploratory studies deemed non-human research by our IRB. The data are governed by data use agreements and not available for other use.

### Anchor-based

The anchor-based MID was calculated based on the relationship of clinic care costs with the 2017 Medicare medical fees for the median (50th percentile) cost for complex clinical visits. The median medical fees for visits of complex (CPT 99,204) and very complex (CPT 99,205) clinic visits were used, because the patient cohort utilized for the low-cost study was comprised of clinically complex patients. The complex visit is defined as a 45-min visit with a median payment of $293, and very complex visit is defined as a 60-min visit with a median payment of $373, a meaningful payment difference of $80 for 15 min. There are no assessments in the literature of clinically meaningful cost differences in hospital admission; thus, only clinic visit cost data were assessed using the anchor-based MID method.

### Distribution-based

MIDs calculated using a distribution-based method were based on Cohen’s cutoffs of 0.2, 0.5, and 0.8 standard deviations (SD) for small, medium, and large effect size (ES), respectively (Cohen 1988). Comparison ES were calculated as a percentage of mean (5%, 10%, 20%) for small, medium, and large ES, respectively. We chose 20% as the maximum change of the cost parameter, because that proportion is commonly used for sensitivity analysis in economic studies to assess value of interventions (Taylor 2009), and our published cost effectiveness models have been shown to be robust to sensitivity analysis employing a 20% change in cost valuation across different clinical trials and country settings (Simpson et al. 2007, 2011). We chose the lower percentages for this parameter to be half and one quarter of the 20% value.

### Consensus-based

MIDs calculated using a consensus-based method were based on the judgement of professionals from various backgrounds in an academic institution that assessed cost evaluations through a questionnaire that was administered via email. The 17 professionals evaluating the questionnaire included 10 faculty (2 finance, 1 health services research, 2 management, 1 policy, 1 informatics, and 1 public health); and seven practitioners (2 hospital administrators, 2 medical practice managers, 1 community health center director and 2 clinical managers). The questionnaire consisted of 4 case scenarios, two low-cost (clinic visits) and two high cost (hospital admissions) scenarios. These scenarios included only mean and standard deviation parameters to mimic costs reported in general research papers. For the visit scenarios, the mean cost reported was $335, and the standard deviation was $237; for the two high-cost (hospital admission) scenarios, the mean cost was $18,400 and the standard deviation was $47,900. Low-cost and high-cost each had one scenario based on a sample size of 100 and one on a sample size of 1000. For each scenario, participants were asked to rate the level of cost savings (based on examples that we derived from MID estimates $17, $35, $47, $67, $80, $120, $190 and $900, $1800, $3600, $9600, $24,000, $38,000; for low-cost and high-cost respectively) as one of the following effect sizes: trivial, small, medium, and large. This approach was not a true Delphi method as there were no additional rounds to form a consensus. However, the results provided narrow bands of estimates and may reflect that an underlying consensus may be reachable with few iterations.

### Power analysis

Power to detect differences were calculated for sample sizes of 100–1000. Power was calculated using 1-sided independent *t *tests for the anchor-based method and 2-sided independent t-tests for the distribution and expert-based methods, all at a 0.05 alpha level. All effect size and power were calculated using log (base 10) transformed costs, as the costs were positively skewed and did not meet normality assumptions. All power calculations were conducted using SAS 9.4 (Cary, NC).

## Results

**Fig. 1:**
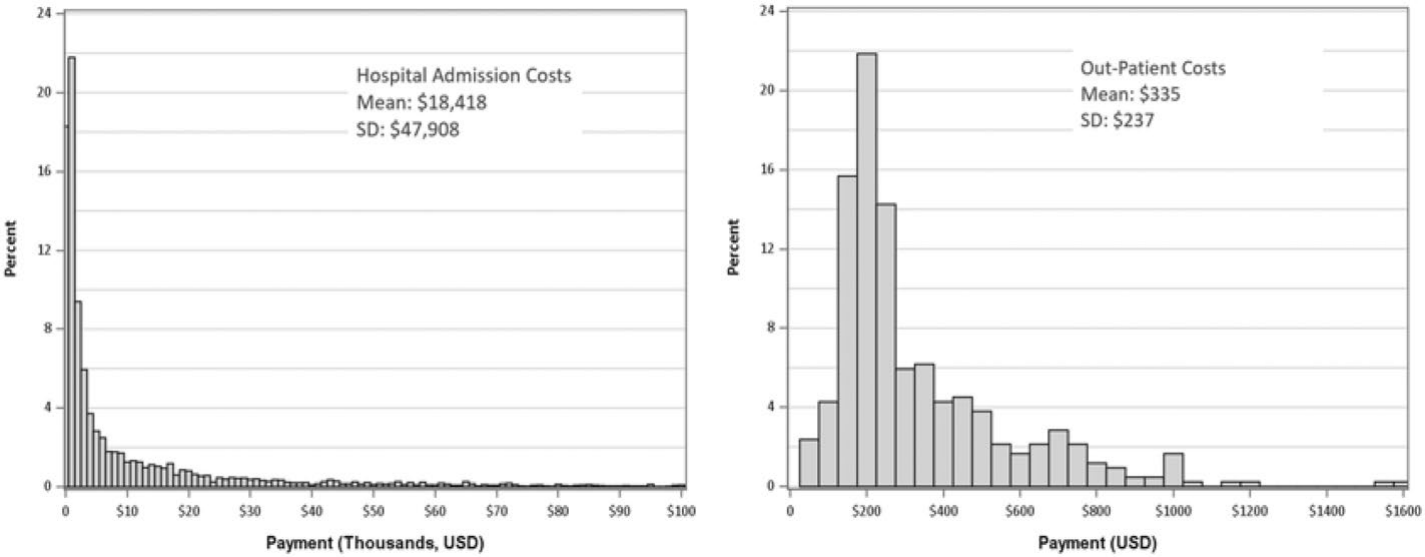
Histogram of hospital costs (left) and clinic visit costs (right) in US dollars

Costs for both the hospital and clinic visit studies had positively skewed distributions (Fig. 1). The hospital admissions study was made up of n = 6427 patients with a mean cost of $18,418 (SD = $47,908) and a median cost of $2,591. The clinic visit study was made up of 60-min visits for n = 421 patients with mean cost of $335 (SD= $237) and a median cost of $237 (Table 1).

**Table 1:** Distribution of cost data for hospital admissions and clinic visits in USD

	N	Mean	SD	Median (Min; Max)
Hospital admissions	6427	$18,418	$47,908	$2591 (1; 1,206,879)
Clinic visit	421	$335	$237	$237 (50; 1617)

### Anchor-based

**Fig. 2:**
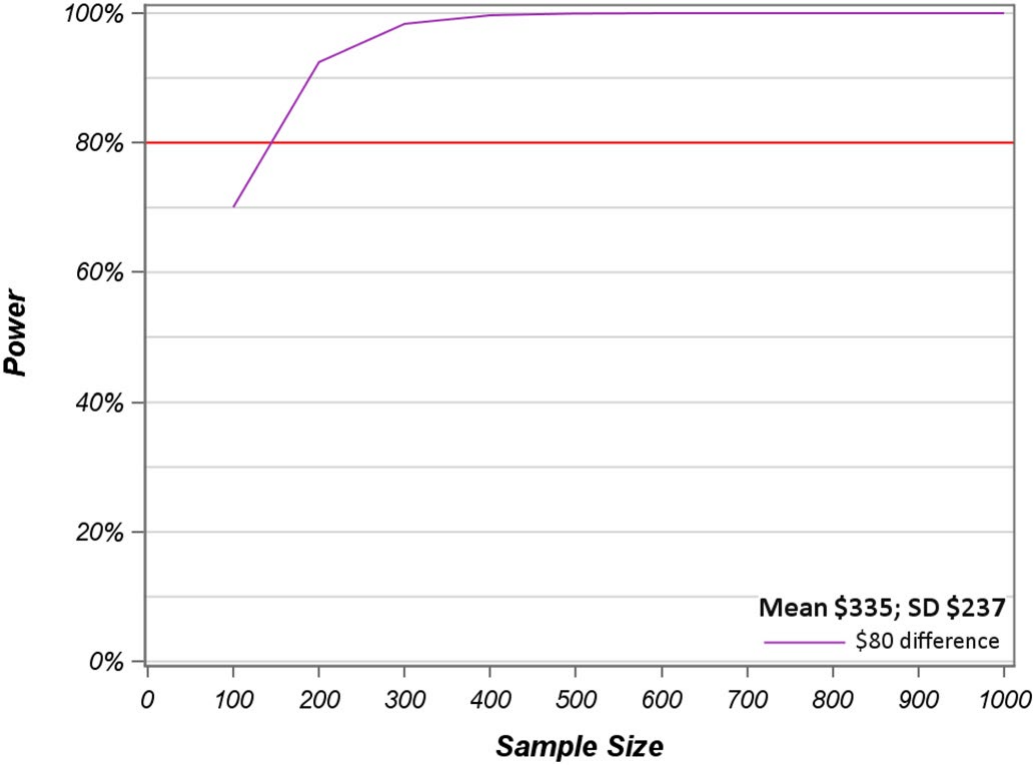
Power calculations of log transformed visit costs for sample sizes 100–1000 for an $80 difference with 80% power level

The anchor-based method using a meaningful payment difference of $80 between the complex 45-min visit and the very complex 60-min visit represents an ES of 0.34 SD and 24% of mean costs. For consistency between graphic displays across methods, Fig. 2 shows the power calculations of the log transformed clinic visit costs for sample sizes 100–1000 based on the difference of $80 under the MID anchor-method with respect to the typically minimally accepted power of 80% as indicated by the red line.

### Distribution-based

**Fig. 3:**
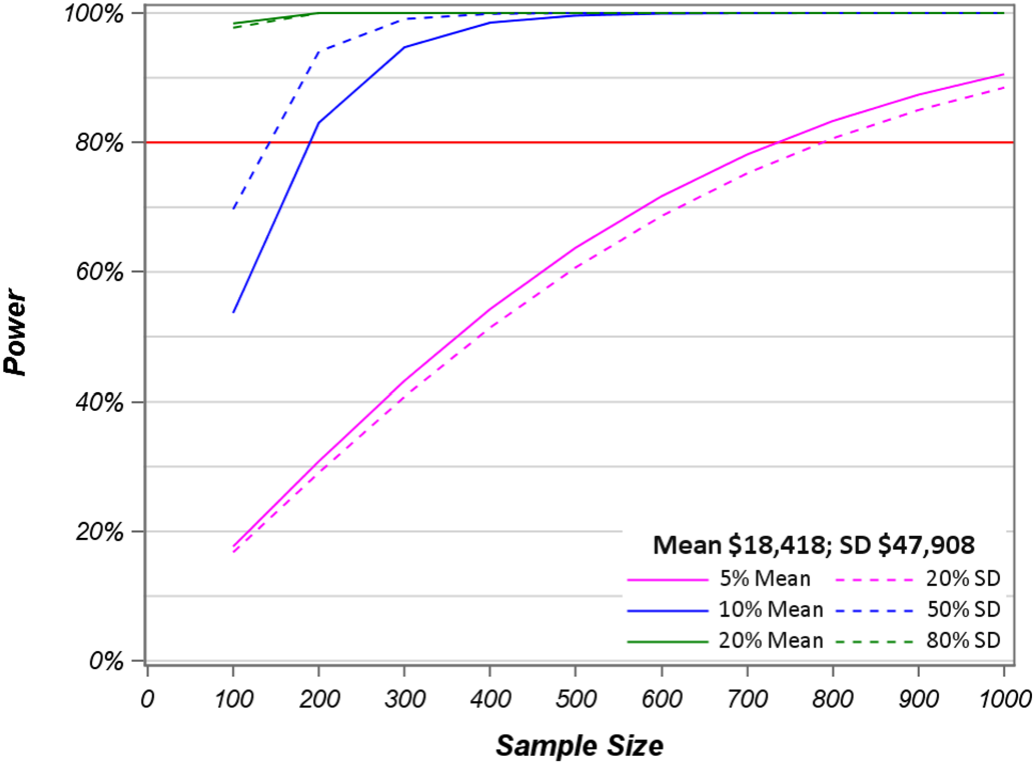
Power calculations of log transformed hospital costs for sample sizes 100–1000 based on percentage of standard deviation and mean with 80% power level

Power calculations of the log transformed hospital admission and clinic visit costs, respectively, were conducted for sample sizes 100–1000 based on the small, medium, and large ES under the MID distribution-based method on standard deviations and percentage of mean costs. For the hospital costs, the power to detect differences for a given sample size based on the distribution-method for MID converges to the power needed to detect differences using the percentage of the mean (Fig. 3) demonstrating the desired convergence across methods. This is encouraging and indicates that the use of percentages and standard deviations to define MIDs may be similar and thus could be used interchangeably or selected in a manner such that they are most relevant or where data availability drives the choice.

**Fig. 4:**
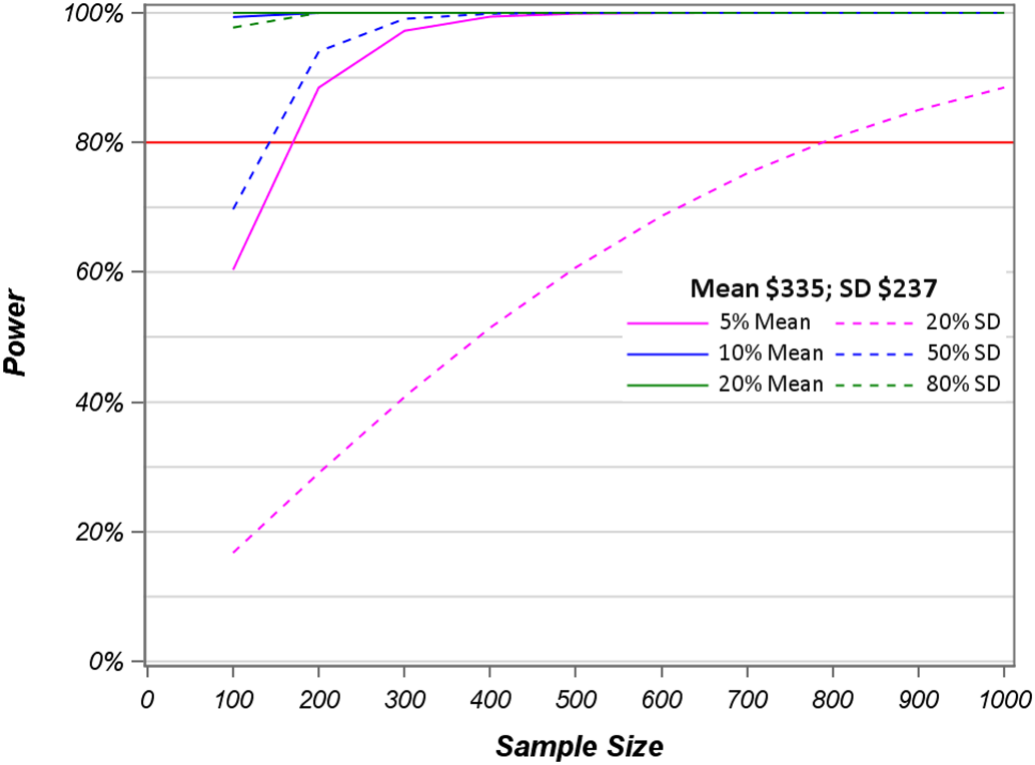
Power calculations of log transformed visit costs for sample sizes 100–1000 based on Cohen’s d suggested cutoffs and percentage of mean with 80% power level

In this high-cost study, we found that the distribution-based method was similar to the method of using a percentage of mean costs. However, under these coefficient of variance assumptions, the sample size needed for a study to be powered to detect a difference using distribution-method of MID is the more conservative calculation when mean cost is small, as is the case with visit costs (Fig. 4).

### Consensus-based

Consensus-based MIDs were calculated based on the 17 questionnaire responses received (85% returned). Two of the missing responses were due to clinicians working on the front line during the COVID-19 pandemic. One missing response was a faculty member. Table 2 shows the average cost savings rated as small, medium, and large effect sizes under each of the four scenarios.

**Table 2:** Results from survey of decision makers in the consensus-based approach: mean (SD) value for survey responses classifying cost savings by effect size given a specified sample size (USD)

Sample size	Low-cost		High-cost	
	100	1000	100	1000
*Effect size*				
Small	$35 (13)	$43 (26)	$1920 (750)	$1575 (402)
Medium	$68 (25)	$68 (31)	$5453 (3242)	$4388 (2681)
Large	$131 (55)	$120 (59)	$18,141 (11,989)	$13,418 (10,977)

The mean cost values from the surveys were calculated as a proportion of the standard deviations and means from the scenarios (Table 3). Both the low cost and high cost proportion of SD are much smaller proportions than the 20%, 50%, and 80% cutoffs for small, medium, and large effect size, respectively, suggested by Cohen. The average rated cost savings among low cost clinic visits as a percentage of mean and SD remained consistent between the two sample size scenarios, indicating that there was a consistent estimate of what comprises a meaningful difference for low cost studies. However, the average rated cost savings among high cost hospital admissions as a percentage of mean and SD remained consistent only among the small and medium ES between the two sample size scenarios. The disagreement is most observable in the large ES as a percentage of the mean; for the smaller sample size, a large ES was considered as much as the mean, whereas, for the larger sample size, it was almost three-quarters of the mean.

**Table 3:** Average survey cost savings as proportion of standard deviation and mean

*N*	Low-cost^a^				High-cost^b^			
	100		1000		100		1000	
	SD (%)	Mean (%)	SD (%)	Mean (%)	SD (%)	Mean (%)	SD (%)	Mean (%)
*Effect size*								
Small	15	10	18	13	4	10	3	9
Medium	29	20	29	20	11	30	9	24
Large	55	39	51	36	38	99	28	73

^a^Mean = $335, SD = $237

^b^Mean = $18,400, SD = $47,900

**Fig. 5:**
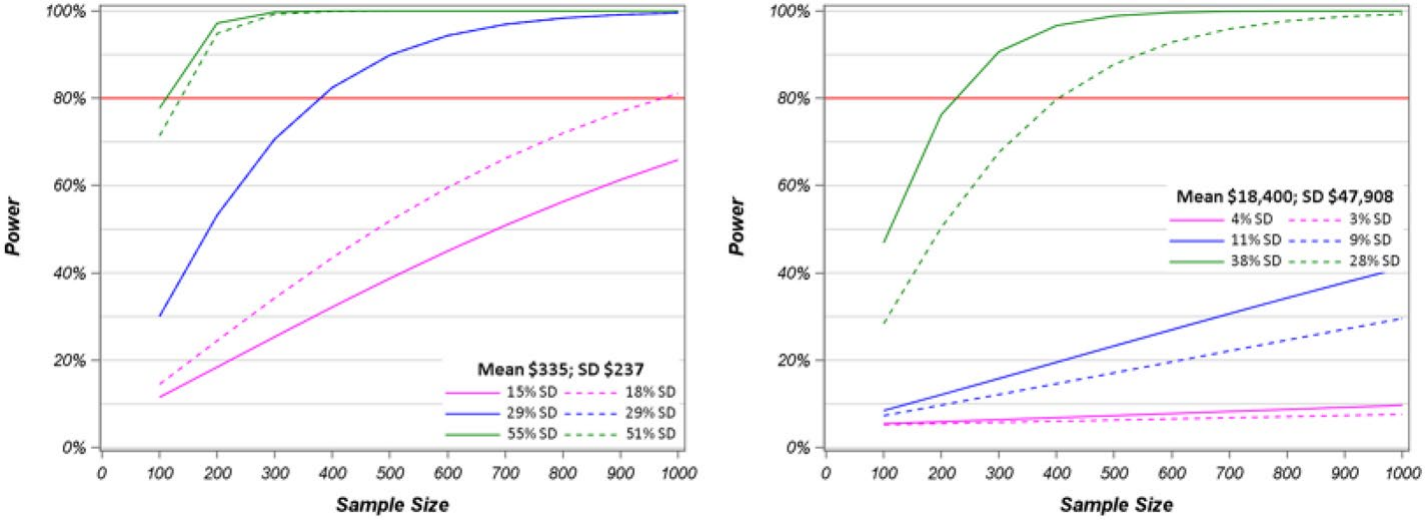
Power calculations of log transformed costs for sample sizes 100–1000 based on rated cost savings as a percentage of SD for low-cost clinic visits (left) and high-cost hospital admissions (right) with 80% power level

The consistency of the proportion of SD between the scenarios of n = 100 and n = 1000 is evident in the power calculations for both low and high-cost as indicated by the closeness of the solid and dotted lines within each color (e.g. green) representing the effect size for small, medium, and large (Fig. 5).

## Summary of findings for the three methods

**Fig. 6:**
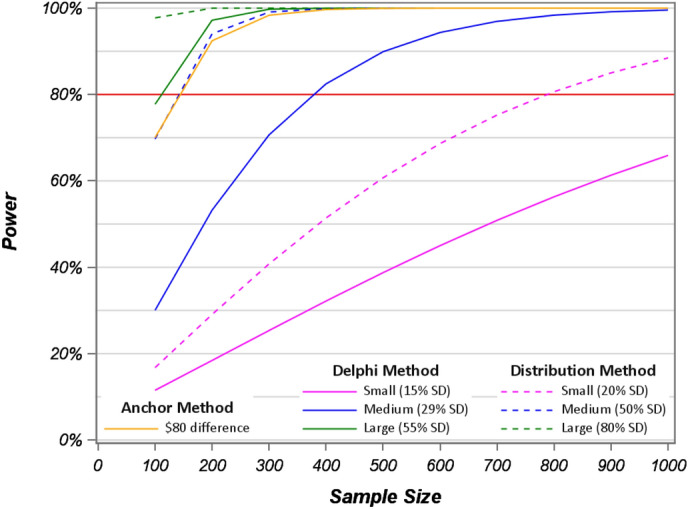
Power calculations of log transformed visit costs for sample sizes 100–1000 based on the anchor, distribution, and Delphi methods with 80% power level

The main results for the three approaches to the specification of MID for cost studies are shown in Fig. 6 below. It appears that the distribution-based method of MID is a usable approach for specifying effect sizes for cost studies. It may be superior to the use of a percent decrease in mean cost for low unit cost studies, because it would guide the researcher towards choosing a more conservative sample size estimate for measuring cost savings. The anchor-based method mapped almost directly to the 50% SD ES of the distribution-based method (Fig. 6) and may prove to be useful if well-defined anchor values are available. We used the difference in payment for a CPT code increment as the anchor. However, we were not able to identify a reasonable anchor value for the hospital admission scenario. This may limit the usefulness of the anchor method for many studies.

**Fig. 7:**
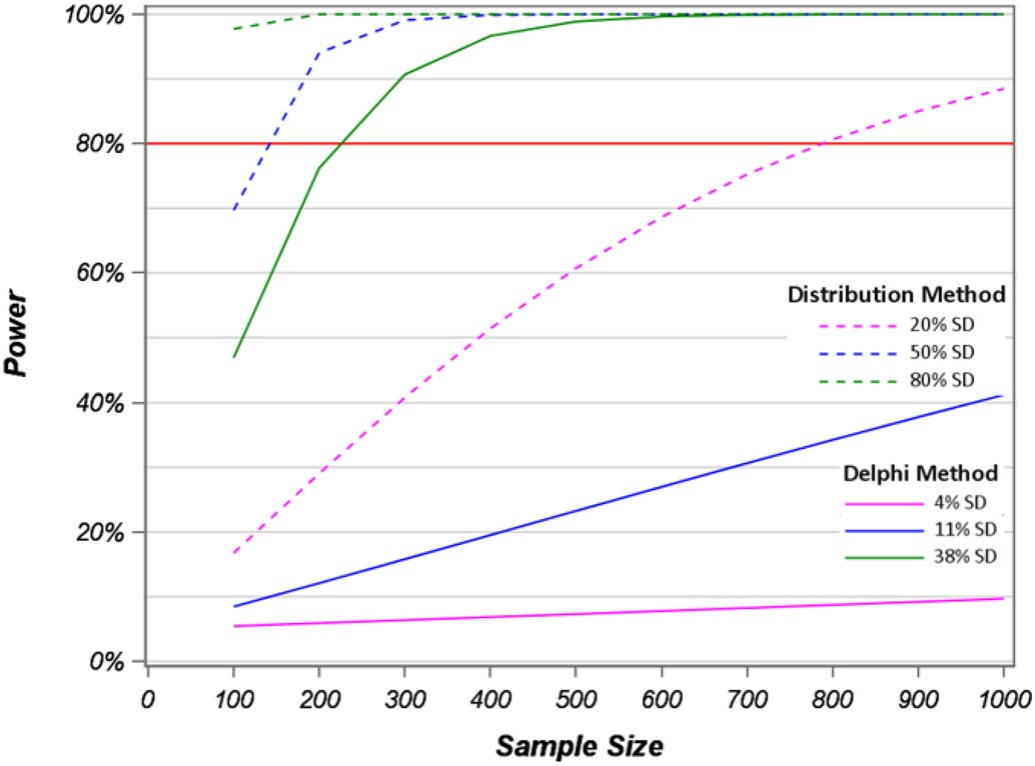
Power calculations of log transformed hospital admission costs for sample sizes 100–1000 based on the distribution and Delphi methods with 80% power level

Several important issues emerged from our study. First, as illustrated by the power curves in Fig. 6, studies that measure savings in low-cost resources, such as medical office visits which have large variances, are rarely adequately powered for testing hypotheses of cost differences if they have < 100 observations, unless they had lower variability than was used in our scenarios. For these types of studies, even a “large” cost difference determined by the distributional method is the only MID specification with > 80% power to detect cost savings. Large sample sizes specified by the Delphi and Anchor methods will require about 200 observations to achieve > 80% power. Studies aiming to detect “medium” sized savings specified by the Delphi method may need at least 400 observations, and small savings may require between 800 and 1400 observations for adequate power. Figure 7 below shows similar patterns for MID estimates for the hospital admission cost data. Only, the very high costs and large SDs make sample size requirements much larger. Indeed, we observed that 1000 observations are inadequate for a study where the expected cost savings are defined by a small or medium size difference, as determined by the Delphi method. Further, to achieve 80% power to find a significant difference for a “small” effect defined by the distribution method may be expected to require about 800 observations.

## Limitations

We found that the Delphi-method MIDs were smaller than the distribution-based MIDs (Figs. 6 and 7); however, the Delphi-method used here was not extended for several rounds to achieve complete consensus among the respondents. Thus, it is possible that the Delphi-method and distribution-based MID could have converged with further iterations. The power and sample size simulations were limited to one study for each hospital admissions and out-patient visits. The Delphi method was conducted on a convenience sample and did not include multiple rounds of the survey’s results to come to a final consensus among respondents. Further simulations need to be conducted to confirm this trend.

## Discussion and conclusions

Our perspective in this paper is that, for health services researchers to make informed decisions about the value of new treatments or process improvements from a population health perspective, we need information on changes in outcomes and costs. Cost is a complex study variable, because it can be viewed from an organizational finance or accounting perspective (fixed and variable costs and budget impact), or from an economic perspective (opportunity cost or cost effectiveness). The value of specifying an MID for cost in a study may be that it will require us to consider cost differences from both perspectives. However, when cost is used to examine value from an economic perspective, it should be defined as a difference in the arithmetic mean cost for the populations treated (Glick et al. 2015, p. 4). This cost measure may be expected to have a non-normal distribution that is skewed with a long heavy right tail, and sometimes with a large number of zero values (Glick et al. 2015, p. 97). Standard deviations for population costs are often equal to or even greater than their mean values. Thus, compared to the distributions for health outcome measures, costs may be expected to require a much greater sample size to achieve sufficient statistical power when compared to the clinical outcomes. This poses practical as well as potentially ethical issues. From a practical perspective, an increased sample size requirement leads to increased study costs and perhaps also longer time before current practice is improved. This potentially deprives patients of better outcomes, which may be considered unethical. Further, increasing study costs may also be considered unethical in a world of scarce resources (Williams 1992).

In studies of program evaluation or organizational quality improvement, we may not be able to increase sample size. This means that understanding the relationship between study power and the definition of a MID specification for cost is important for study planning. Given the high likelihood that the cost measure in a study greatly affects study power, we compare our MID specification on this metric, not because it is the “best” comparator, but because it may be expected to be the greatest constraint on the study design and should be considered early in the study planning phase. In addition, the use of study power as a metric for comparing different definitions of an MID illustrates to readers the complex relationships that exist between the recommended measures of cost (arithmetic mean and SD), sample size, and a study’s power to make inferences about the value gained by the intervention (Glick et al. 2015, p. 110). There are no firm criteria for choosing a superior method for MID. Indeed, it may be desirable to use more than one approach and examine convergence. However, three criteria should be considered: (1) the chosen method should be relevant to decision makers, (2) the method should fit the type of preliminary cost data available prior to the study (e.g. cannot use the anchor method if cost data is not available for the anchor, or if an anchor that is acceptable to the major stake holders cannot be identified), and (3) the method should reflect current benchmarks (if any) that are available for the condition in the literature.

Additional research must be conducted to determine if the Delphi-method, when fully implemented, agrees with the distribution and anchor-based method findings. Based on the limited results of this small study, it appears that the anchor-based method, while logical and simple to implement, future research should focus on identifying appropriate anchors. It may be the case that it becomes easier to define anchors as we begin to think in-depth about defining cost MIDs. Our thinking has evolved over the time of this study. When we planned this work, we could not think of a good anchor for hospital admissions. However, now, after much discussion, we recommend that future studies of the MID for hospital cost explore the potential value of using Diagnosis-Related Group (DRG) incremental payment differences as MID anchors for hospital admissions. We therefore recommend that all three methods for defining the for MID for costs be explored further and be examined for convergence. Other issues of importance have emerged as a result of this exploratory work. From the responses to our Delphi survey, it appears that it may be important to examine if the absolute value of costs affect decision makers perception of cost savings. Health services research cost studies have a number of different audiences, and the MIDs may vary between decision-making groups. Despite the low sample size, we observed potential clustering of responses within similarly trained groups. MIDs defined as “Big” by clinicians, accountants, and administrators may differ. Thus, future studies should explicitly examine if MIDs differ by current responsibility or professional training of respondents to Delphi surveys.

## Data Availability

Access to data is limited by data usage agreement so data is not available.
